# Repair of chromosomal breaks by NHEJ

**DOI:** 10.18632/oncotarget.4593

**Published:** 2015-06-23

**Authors:** Dylan A. Reid, Eli Rothenberg

**Affiliations:** Department of Biochemistry and Molecular Pharmacology, New York University School of Medicine, New York, NY, USA

**Keywords:** Chromosome Section

Chromosomal double stranded breaks (DSBs) are regarded as the most cytotoxic form of DNA damage, and occur as a result of normal cellular processes, such as DNA replication, as well as in response to ionizing radiation, and chemotherapeutics. These breaks must be repaired to ensure genomic stability and viability. In vertebrates, nonhomologous end joining (NHEJ) is the main pathway for repair of DSBs, and is active throughout the cell cycle. According to common measures for the rate of occurrence of DSBs in cell culture, an estimated 10^12-14^ DSBs are repaired in the human body each day [1, 2]. NHEJ is therefore vital for maintaining genomic stability by suppressing gross chromosomal translocations and for offsetting senescence, apoptosis, and the aging process. Deficient NHEJ also often manifests as immune system deficiency due to the role of NHEJ in repairing DNA breaks generated in V(D)J and immunoglobulin class-switch recombination.

The NHEJ complex is a multi-protein machine whose composition depends on the complexity of the DSB. The core NHEJ complex is composed of the Ku70/86 (Ku) heterodimer, the DNA dependent protein kinase catalytic subunit (DNA-PKcs), XRCC4, XLF, and DNA Ligase IV (LigIV). The proteins of the core complex recognize DSBs, recruit accessory NHEJ factors, maintain synapsis and complete the ligation of broken ends. Initial recognition of DSBs is performed by Ku, which encircles DNA ends and serves as a molecular scaffold for recruiting DNA-PKcs, XRCC4, XLF, and LigIV. Beyond their interaction with Ku, the core factors interact with one another and with the accessory NHEJ factors. XRCC4 and LigIV are tightly associated, forming the LX complex, as well as associating with XLF.

Despite the importance of the NHEJ pathway, how these factors organize at DSB sites to form a functional repair complex and how the broken ends are brought together remained largely undefined. In our recent study, we applied single-molecule fluorescence imaging methods to reveal important organization and functional information about NHEJ in human cells [3]. Using super-resolution fluorescence localization microscopy, which offers a ten-fold improvement in resolution compared to confocal, we identified NHEJ filaments formed by XRCC4/XLF/LigIV *in vivo* and *in vitro*. Other groups had previously postulated that NHEJ proteins form filaments, though evidence for their existence *in vivo* had not been previously forthcoming. We showed that Ku is localized at either the one end or towards the center of the filament. We show that these structures become more prevalent with damage, and that their frequency of occurrence decreases with recovery time.

To monitor how ends are positioned during synapsis in real-time, we developed single-molecule FRET assays that can readily distinguish between paired (but non-ligated) ends, and ends that are ligated across one or both strands. These assays enabled us to monitor initial pairing and subsequent alignment and ligation, as well as the overall yield of pairing and ligation. By varying the NHEJ factors added and scoring for the number of paired complexes observed, we found that Ku/LX/XLF provided the highest pairing yield, and that LX/XLF promotes pairing in the absence of Ku. Furthermore, these experiments showed that in the initial pairing configuration, the DNA molecules are not positioned in an end-to-end fashion, but rather in an adjacent configuration, suggesting that pairing is mediated by filament-filament contacts. These NHEJ filaments provide a unique interaction landscape for DSBs repair, ensuring that broken ends will make contact and undergo appropriate repair.

Our observations provide a new and refined model for DSB repair by NHEJ, which is considerably different from the previous simplistic “text book” model. In contrast to the established view whereby broken ends are simply held and ligated together by protein complexes, our findings show that the ligation complex forms filaments and that these mediate both the pairing and ligation steps. These findings also outline the advantage of using innovative single-molecule fluorescence imaging methods for defining the previously unattainable physical and functional form of DNA repair complexes. Further studies using these methods will provide us with critical insights into the molecular mechanism of a variety of critical pathways in healthy cells and how these pathways are perturbed in disease, and afford new powerful assays for diagnostics, drug screening and personalized therapy.
